# Roles of Two Phytoene Synthases and Orange Protein in Carotenoid Metabolism of the β-Carotene-Accumulating *Dunaliella salina*

**DOI:** 10.1128/spectrum.00069-23

**Published:** 2023-04-06

**Authors:** Ming-Hua Liang, Shan-Rong Xie, Jv-Liang Dai, Hao-Hong Chen, Jian-Guo Jiang

**Affiliations:** a Guangdong Provincial Key Laboratory of Biotechnology for Plant Development, Guangzhou Key Laboratory of Subtropical Biodiversity and Biomonitoring, Institute of Ecological Science, School of Life Sciences, South China Normal University, Guangzhou, China; b School of Food Science and Engineering, South China University of Technology, Guangzhou, China; Ocean University of China

**Keywords:** *Dunaliella salina*, phytoene synthase, orange protein, carotenoid, protein interaction, overexpression

## Abstract

Phytoene synthase (PSY) is a key enzyme in carotenoid metabolism and often regulated by orange protein. However, few studies have focused on the functional differentiation of the two PSYs and their regulation by protein interaction in the β-carotene-accumulating *Dunaliella salina* CCAP 19/18. In this study, we confirmed that DsPSY1 from *D. salina* possessed high PSY catalytic activity, whereas DsPSY2 almost had no activity. Two amino acid residues at positions 144 and 285 responsible for substrate binding were associated with the functional variance between DsPSY1 and DsPSY2. Moreover, orange protein from *D. salina* (DsOR) could interact with DsPSY1/2. DbPSY from *Dunaliella* sp. FACHB-847 also had high PSY activity, but DbOR could not interact with DbPSY, which might be one reason why it could not highly accumulate β-carotene. Overexpression of *DsOR*, especially the mutant *DsOR^His^*, could significantly improve the single-cell carotenoid content and change cell morphology (with larger cell size, bigger plastoglobuli, and fragmented starch granules) of *D. salina*. Overall, DsPSY1 played a dominant role in carotenoid biosynthesis in *D. salina*, and DsOR promoted carotenoid accumulation, especially β-carotene via interacting with DsPSY1/2 and regulating the plastid development. Our study provides a new clue for the regulatory mechanism of carotenoid metabolism in *Dunaliella*.

**IMPORTANCE** Phytoene synthase (PSY) as the key rate-limiting enzyme in carotenoid metabolism can be regulated by various regulators and factors. We found that DsPSY1 played a dominant role in carotenogenesis in the β-carotene-accumulating *Dunaliella salina*, and two amino acid residues critical in the substrate binding were associated with the functional variance between DsPSY1 and DsPSY2. Orange protein from *D. salina* (DsOR) can promote carotenoid accumulation via interacting with DsPSY1/2 and regulating the plastid development, which provides new insights into the molecular mechanism of massive accumulation of β-carotene in *D. salina*.

## INTRODUCTION

Carotenoids are a group of yellow, orange-red. or red pigments found in plants, algae, fungi, and bacteria, which can give various colors to fruits and vegetables, flowers, animal feathers, and shellfish. More than 700 carotenoids have been identified. They also play key roles in the light absorption and transfer of light energy, protection of chlorophyll molecules, promotion of photomorphogenesis, synthesis of hormones, and stress response (1, 2). Meanwhile, carotenoids such as β-carotene, α-carotene, and β-cryptoxanthin are the main sources of vitamin A in the human body, which play vital roles in vision and the immune system. β-carotene can be used as natural colorants, animal feed supplements, and nutraceuticals, and it is good for health benefits with anti-oxidation, improving immune capacity, protecting eyesight and skin, and anticancer and anti-aging activities (3).

In plants, the regulatory mechanisms of carotenoid accumulation are complex, which can be regulated by the synthesis, degradation, and storage, affected by transcription, transcription factors, and protein levels, epigenetic modification, etc., and also influenced by different growth or developmental stages, hormones, and environmental stresses (4, 5), and each process can cause changes in carotenoid levels. Phytoene synthase (PSY) is the first key rate-limiting enzyme in carotenogenesis, catalyzing the generation of the first carotenoid, C_40_ phytoene, from two molecules of C_20_ geranylgeranyl pyrophosphate (GGPP). Regulation of *PSY* can alter the carotenoid levels (5 to 7). The *PSY* gene has often been used as the first choice in carotenoid genetic engineering, and its rational design can be used for production of carotenoid-rich crops (7, 8). The *PSY* gene from the daffodil, which was introduced into rice endosperm to obtain the first generation of “golden rice” (2000) by genetic engineering, had low catalytic efficiency, and its carotenoid content was only about 1.6 μg/g (9). In the second generation of “golden rice” (2005), the more active *PSY* gene from maize was introduced, which increased the total carotenoids up to 23-fold (maximum 37 μg/g) (10). However, few comparative studies have focused on the functional activity, gene expression, and regulatory mechanism of the two PSYs present in some algae, and the key amino acid sites affecting PSY enzyme activity in algae are unclear.

The enzyme activity of PSY can be affected by the interaction of regulatory proteins. It is reported that stay-green protein (SGR) from tomatoes (SlSGR1) can negatively regulate the activity of SlPSY1 by interacting with SlPSY1, thus affecting the accumulation of carotenoids in tomato fruits (11). In addition, orange proteins (ORs) from plants can interact with PSY and regulate plastid development (12, 13). In recent years, overexpression of *OR* genes from cauliflower, Arabidopsis thaliana, and other plants into cauliflower, potato, tomato, and other plants can effectively improve the activity of PSY and regulate plastid development (increase plastid size and plastid number, induce plastid differentiation, or promote the development of chromoplast, etc.), thus significantly improving the accumulation of carotenoids, especially β-carotene (14 to 17). Furthermore, OR^His^ (an arginine to histidine substitution) can still interact with PSY, and overexpression of *AtOR^His^*, *SbOR^His^*, or *CmOR^His^* genes can further increase the carotenoid content in the chromoplast of *Arabidopsis*, sorghum, tomato, or melon (16 to 18). Notably, the discovery of the *OR* gene provides a new and effective way to improve carotenoid content and nutritional quality in food crops and has a good application prospect. However, recently, overexpression of *OsOR* in Nipponbare rice was found to negatively regulate carotenoid accumulation, improve tillering number, and reduce stress resistance (19). For mainly algae Chlamydomonas reinhardtii, the accumulation of carotenoids can also be improved by overexpression of the *PSY* or *Reinhardtian* gene (20 to 23). Nevertheless, how the algal OR protein regulates PSY activity has not been reported. Whether there are regulatory proteins that interact with the PSY enzyme in algae is still rarely reported.

The halotolerant green alga *Dunaliella salina* CCAP 19/18 can accumulate massive β-carotene in chloroplasts in the form of plastoglobuli, especially under stress conditions, which has been considered a model for studying the mechanism of carotenoid metabolism and osmotic regulation and used for commercial production of natural carotenoids (24 to 26). The cells of *D. salina* CCAP 19/18 can change from the initial green to orange at the later period with β-carotene (>80% of the total carotenoid) as the main carotenoid (25, 27). However, some *Dunaliella* species belong to non-β-carotene-accumulating strains, such as Dunaliella tertiolecta CCAP 19/6B (28) and *Dunaliella* sp. FACHB-847 (25, 29). The cells of *Dunaliella* sp. FACHB-847 can maintain a green color with high chlorophyll content and lutein (>50% of the total carotenoid) as the main carotenoid, which has been considered the potential source of lutein (25, 30). In this study, we compared the functional activities of PSYs from *D. salina* CCAP 19/18 and *Dunaliella* sp. FACHB-847, and the interaction of OR protein with PSY was investigated in these two strains. In addition, we would find out the key amino acid sites determining the functional variance between the two PSYs (DsPSY1/2) as well as the role of DsOR in carotenoid metabolism in the β-carotene-overproducing *D. salina.*

## RESULTS AND DISCUSSION

### Cloning and functional identification of two phytoene synthase genes from *D. salina*.

**FIG 1:**
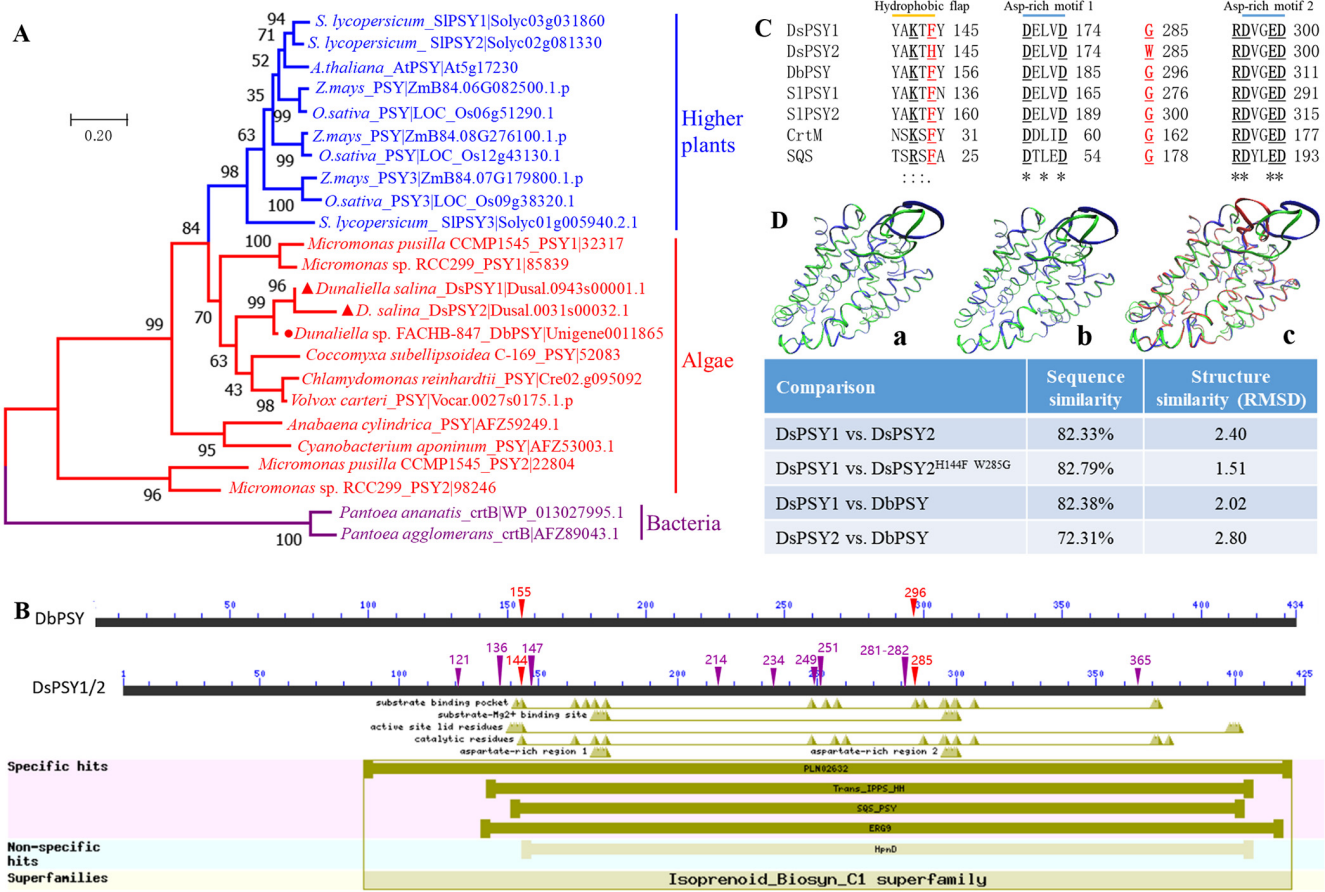
Molecular characterization of phytoene synthases from *Dunaliella salina* and *Dunaliella* sp. FACHB-847. (A) Phylogenetic analysis of PSYs from different species; (B) conserved domains in DsPSY1/2 and DbPSY; (C) conserved core structures, including hydrophobic flap and two Asp-rich motifs between PSY, CrtM, and SQS enzymes. Ds, *D. salina*; Db, *Dunaliella* sp. FACHB-847; Sl, *Solanum lycopersicum*; SlPSY1, UniProtKB/Swiss-Prot: P08196; SlPSY2, UniProtKB/Swiss-Prot: P37273; CrtM from Enterococcus hirae, PDB: 5IYS; SQS from Homo sapiens, PDB: 1EZF. (D) Analysis of sequence and structure similarities between different PSYs. (a) Superposition of DsPSY1 (blue) and DsPSY2 (green); (b) superposition of DsPSY1 (blue) and DsPSY2^H144F W285G^ (green); (c) superposition of DsPSY1 (blue), DsPSY2 (green), and DbPSY (red). The parameters of sequence and structure similarities between different PSYs are shown in the table.

According to the draft nuclear genome sequence of *Dunaliella salina* strain CCAP 19/18 at the phytozome website (31), two phytoene synthase genes, with gene identifiers with Dusal.0943s00001 and Dusal.0031s00032, were identified, and designated *DsPSY1* and *DsPSY2*, respectively. Their full-length coding sequences were 1,278 bp with 425 amino acids (aa). Predicted by Plant-mPLoc, DsPSY1 and DsPSY2 were localized in the chloroplast. The length of the predicted chloroplastic transit peptides of DsPSY1/2 were 40 and 41 amino acid residues at the N terminals, respectively. In addition, we also cloned the *PSY* gene from *Dunaliella* sp. FACHB-847 (*DbPSY*) according to our previous transcriptome data, with the full-length coding sequence (CDS) of 1,305 bp encoding 434 aa (32). The length of the predicted chloroplastic transit peptide of DbPSY was 52 amino acid residues at the N terminals. The protein sequence of DsPSY1 displayed high identities with DbPSY (82.38%) and DsPSY2 (82.33%), with the main sequence differences in the transit peptide regions. *Dunaliella* PSYs (DsPSY1/2 and DbPSY) showed high homologies with PSYs from other green algae and higher plants, and showed ~50% identities with cyanobacterial crtB and ~30% identities with bacterial crtB (Fig. 1A). Many plants have 2 to 3 *PSY* homologous genes with tissue expression specificity; for example, tomato has 3 *PSY* genes, among which *SlPSY1* is specifically expressed in fruits and flowers, and SlPSY2 is mainly expressed in leaves (33). Dicot *PSY3* including *SlPSY3* is strongly expressed upon root colonization by symbiotic arbuscular mycorrhizal fungi for apocarotenoid formation (34), distinct from *PSY3* genes in the Poaceae, which can be induced by abiotic stress such as salt and drought (35). However, there is only one *PSY* gene in Arabidopsis thaliana. In contrast, most algae, including *Dunaliella* sp. FACHB-847, possess only one *PSY* gene (36); however, two *PSY* homologous genes were found in some algae, such as *D. salina* CCAP 19/18, *Ostreococcus*, and *Micromonas* based on the phytozome website (31, 37). Algal *PSY* genes have been found to be induced by multiple stress factors such as high light intensity, blue light, salt stress, and nutrient deficiency (36, 38, 39).

**FIG 2:**
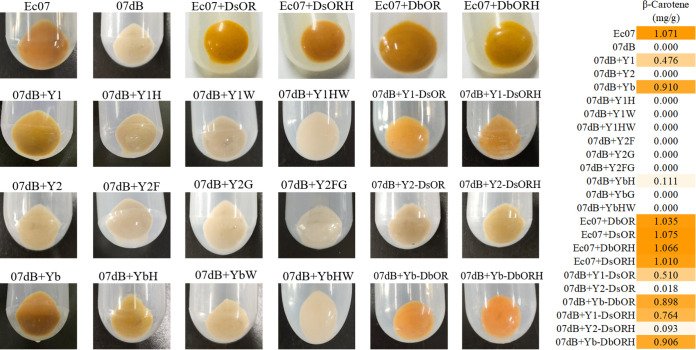
Functional identification of DsPSY1/2 and DsOR in β-carotene-producing E. coli. The engineered E. coli strains were induced by 1.0 mM IPTG when OD_600_ was 0.6, and cultivated at 30°C, 200 rpm for 48 h for carotenoid extraction and analysis. The colors and the β-carotene levels of the engineered E. coli are shown.

Furthermore, we used color complementation in the carotenoid-producing E. coli platform to investigate the functional activity of DsPSY1/2 compared with the activity of DbPSY. The orange-colored Ec07 (harboring pAC-BETA) could result in the accumulation of β-carotene (40) (Fig. 2), whereas no carotenoids were observed in the 07 dB strain (carrying pAC-EIYdB) with the disruption of the bacterial *crtB* gene encoding phytoene synthase. When *DsPSY1/2* and *DbPSY* without chloroplast transit peptides were overexpressed into 07 dB, respectively, the resulting strain 07 dB+Yb could accumulate comparable β-carotene with Ec07, and 07 dB+Y1 could lead to the considerable accumulation of β-carotene, but produced lower β-carotene than 07 dB+Yb. However, almost no β-carotene could be detected in 07 dB+Y2 (Fig. 2). This indicated that DsPSY1 and DbPSY had functional activity of PSY, but DsPSY2 had almost no PSY activity. Apparently, DsPSY1 was less effective in promoting carotenoid biosynthesis than DbPSY. *Dunaliella* sp. FACHB-847 was rich in lutein, which could be due to this efficient enzyme activity of DbPSY, the bifunctional lycopene β- and ε-cyclases, and multiple pathways for lutein biosynthesis by carotene hydroxylase as well as the high expression of carotenogenic genes (25, 30, 36).

### Two key amino acid residues associated with the functional variance between DsPSY1 and DsPSY2.

Searched by NCBI-conserved domains, DsPSY1/2 and DbPSY possessed multiple distinct conserved domains, such as the substrate binding pocket, substrate-Mg^2+^ binding site, active site lid residues, catalytic residues, and two aspartate-rich regions (Fig. 1B). The protein sequences of DsPSY1/2 were highly homologous, but their catalytic efficiencies were distinctly different (Fig. 2). In tomato, fruit-specific SlPSY1 was reported to exhibit significantly weaker carotenogenic activity than green tissue-specific SlPSY2, and it was proposed that a neighboring aromatic-aromatic amino acid combination (Phe-135 and Asn-136 in SlPSY1, Phe-159 and Tyr-160 in SlPSY2) governed activity divergence between these two PSYs from tomato (33). Therefore, we speculated some key amino acid sites leading to the differentiation of functional activity of DsPSY1/2.

In order to elucidate the reason for differential catalytic activity of DsPSY1/2, we first predicted a total of 13 amino acid positions (at 121, 136, 144, 147, 214, 234, 249, 251, 281, 282, 284, 285, and 365) that led to harmful mutations of DsPSY1 with high possibilities, through the PolyPhen-2 website (http://genetics.bwh.harvard.edu/pph2/) (Fig. 1B). We also used DbPSY for mutation experiments by testing the change in catalytic activity by amino acid substitution. Combined with the result of sequence alignment and conserved domain, two possible key amino acid sites (F144 and G285 in DsPSY1; H144 and W285 in DsPSY2; F155 and G296 in DbPSY) correlated with substrate binding pocket sites were used for further mutation experiments (Fig. 1B). Accordingly, by site-directed mutagenesis, we generated mutants DsPSY1^F144H^, DsPSY1^G285W^, DsPSY1^F144H G285W^, DsPSY2^H144F^, DsPSY2^W285G^, DsPSY2^H144F W285G^, DbPSY^F155H^, DbPSY^G296W^, and DbPSY^F155H G296W^ for mutational analysis by testing catalytic activity. The mutated PSYs coupled with pAC-EIYdB were expressed in E. coli BL21(DE3). As shown in Fig. 2, 07 dB+Y1H, 07 dB+Y1W, and 07 dB+Y1HW carrying the mutant forms of F144H or/and G285W of DsPSY1 could greatly reduce the cell color with almost no detectable β-carotene content, as did 07 dB+YbW and 07 dB+YbHW. F155H in DbPSY also largely decreased the β-carotene content. Although the amino acid substitutions H144F and W285G as well as their double mutation in DsPSY2 did not result in β-carotene accumulation, it was sufficient to suggest that the two amino acid residues at positions 144 and 285 critically determined the catalytic activity difference between DsPSY1 and DsPSY2.

**FIG 3:**
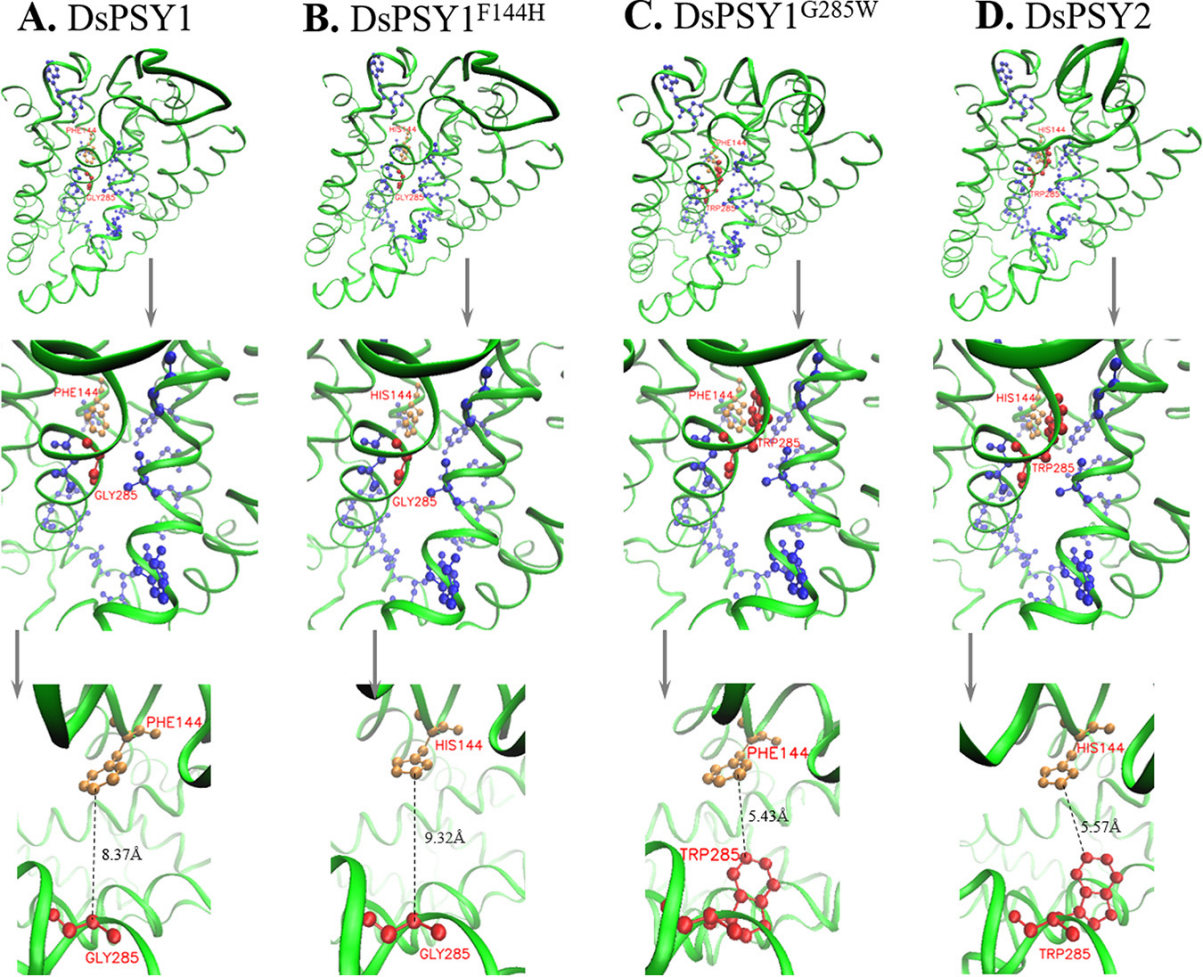
Three-dimensional protein structures of the wild-type DsPSY1/2 and mutated DsPSY1. (A) DsPSY1; (B) DsPSY1^F144H^; (C) DsPSY1^G285W^; (D) DsPSY2; The backbone and sidechain of specific amino acid residues responsible for substrate binding pocket are displayed, including residue at 144 labeled in orange, residue at 285 in red, and other specific residues in blue.

Similar to PSY that catalyzes head-to-head condensation of GGPP to generate phytoene, dehydrosqualene synthase (CrtM) and squalene synthase (SQS) catalyze head-to-head condensation of two molecules of farnesyl pyrophosphate to form dehydrosqualene and squalene, respectively (33). PSY proteins shared conserved core structures such as the hydrophobic flap and Asp-rich motif 1/2 with CrtM and SQS (41) (Fig. 1C). Here, we analyzed the protein structures of *Dunaliella* PSY proteins by homology modeling using SWISS-MODEL based on the crystal structure of CrtM from Enterococcus hirae ATCC 9790 (PDB: 5IYS) (Fig. 1D and 3). DsPSY1/2 and DbPSY with high sequence similarities had a similar protein structure with RMSD (root mean square deviation) lower than 3. The higher sequence similarity, the lower the RMSD and the better the protein superposition (Fig. 1D).

As shown in Fig. 3, the backbone and sidechain of amino acid residues responsible for substrate binding pockets (looking like “pockets”) were shown in the protein structures, which were highly similar with other PSY enzymes, CrtM, and squalene synthase (SQS) (41, 42). F144 and G285 in DsPSY1 were highly conserved with many PSYs from other green algae and higher plants, of which F144 played a critical role in substrate binding, active site, and catalysis. G285 was also particularly important for substrate binding (Fig. 1B). F144 in DsPSY1 was conserved with one of the neighboring aromatic-aromatic amino acids that determined the activity divergence between the two PSYs from tomato (33). Interestingly, the spatial position of residues at 144 and 285 of DsPSY1/2 in 3D protein structure was almost the opposite, and located in the opening of the substrate binding pocket. For DsPSY1 (Fig. 3A), when F144 containing a 6-carbon benzene ring was mutated into H144 with an imidazole side chain, the longer spatial distance was observed between residues at 144 and 285 (Fig. 3B), which may lead to the loose binding between PSY enzyme and substrate, thereby reducing the catalytic efficiency. When G285 with simple molecular structure in DsPSY1 was mutated into W285 with an indole ring, much shorter spatial distance was observed between residues at 144 and 285 (Fig. 3C), which may hinder the substrate binding and result in the great reduction in catalytic activity. Similarly, the narrowing of the opening of the substrate binding pocket (Fig. 3D) may account for the near inactivation of DsPSY2. Besides, multiple amino acid residues at positions 121, 136, 147, 214, 234, 249, 251, 281, 282, 284, and 365 that were potentially harmful for the PSY catalytic activity were present in DsPSY2, which led to a near loss of enzyme activity. It was reported that the amino acid replacement of P192L led to the misfolding of SlPSY1 protein and affected its activity, thus reducing the accumulation of lycopene and other carotenoids in tomato fruits (43). An amino acid mutation of A191D in the *PSY* gene from cassava could promote the accumulation of provitamin A carotenoids (6). Overall, the key amino acid residues have a great impact on PSY enzyme activity, and the rational design of the *PSY* gene can be used for production of carotenoid-rich crops (7, 8).

### Cloning and characterization of orange protein from *D. salina*.

**FIG 4:**
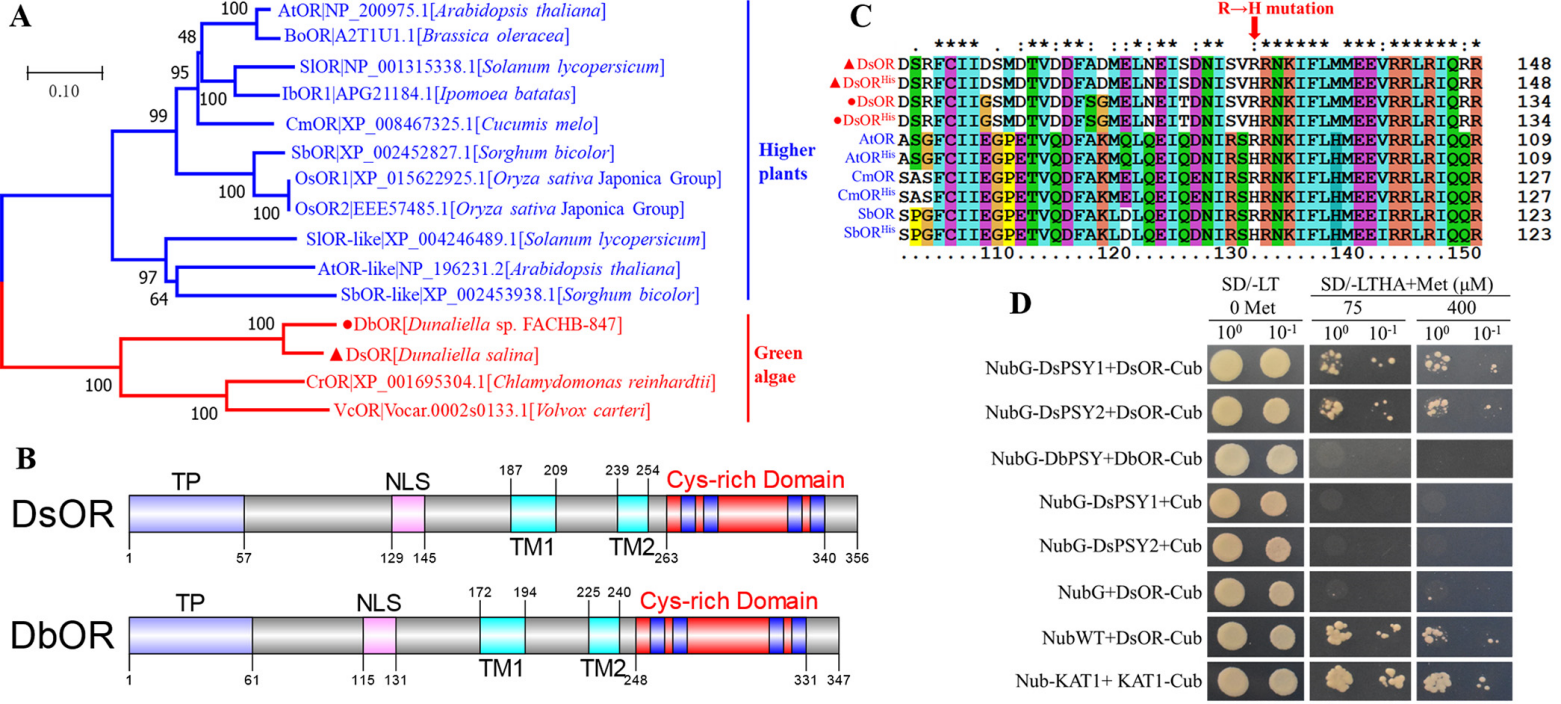
Molecular characterization of *Dunaliella* OR proteins and their interaction with PSY. (A) Phylogenetic analysis of OR proteins from plants and green algae; (B) domain analysis of DsOR and DbOR. TP, transit peptide; NLS, nuclear localization signal; TM1 and TM2, transmembrane helices. Four repeats of the CxxCxxxG signatures at the C terminal are shown in blue in the Cys-rich domain. (C) The mutation of highly conserved arginine to histidine of OR proteins. Ds, D. salina; Db, *Dunaliella* sp. FACHB-847; At, A. thaliana; Cm, Cucumis melo; Sb, Sorghum bicolor. (D) Mating-based split-ubiquitin system (mbSUS) of yeast two-hybrid assay showing that DsOR interacted with DsPSY1/2. Wild-type Nub (NubWT) and the mutant Nub (NubG) vectors acted as the positive and negative controls of NubG-DsPSY1/2, respectively. Cub served as the negative control for DsOR-Cub. Yeast cells coexpressing Nub-KAT1 and KAT-Cub acted as the positive control of the mbSUS assay. SD/-LT 0 Met, synthetically defined medium (SD) lacking leucine, tryptophan, and methionine (SD/-Leu/-Trp/-Met); SD/-LTHA+Met, SD medium lacking leucine, tryptophan, histidine, and adenine (SD/-Leu/-Trp/-His/-Ade) containing methionine.

According to the genome data of *D. salina*, only partial CDS of the *orange* gene from *Dunaliella salina* (designated *DsOR*) could be identified with the gene identifier with Dusal.0142s00018. Based on our transcriptome data of *D. salina*, the full-length CDS of *DsOR* with 1,071 bp encoding 356 aa could be cloned. Transcriptome sequencing of *Duneliella* sp. FACHB-847 found that the full-length CDS of *DbOR* was 1,044 with 347 aa (32), which displayed 75.76% identities with DsOR. The protein sequence of DsOR showed 50 to 76% identities with ORs from other green algae and higher plants (Fig. 4A). DsOR belonged to the DnaJ-like zinc finger domain-containing protein family and DnaJ_zf superfamily, and shared the highly conserved quadruple repeat of the CxxCxxxG signatures at the C terminal (Fig. 4B). Analyzed by Phyre2, DsOR and DbOR were predicted as a membrane protein with two transmembrane helices (Fig. 4B). DsOR was predicted to be localized in the chloroplast and mitochondrion by Plant-mPLoc prediction, and localized in the nucleus by LOCALIZER prediction. In plants, the multifunctional OR proteins have been reported to be localized in different subcellular localizations, including nucleus and plastids (44, 45). Predicted by LOCALIZER, a putative chloroplast transit peptide and a nuclear localization signal (RRNKIFLMMEEVRRLRI) were present in DsOR and DbOR (Fig. 4B). An arginine to histidine “golden SNP” has been shown to contribute to large amounts of carotenoids (18). Accordingly, a highly conserved arginine at positions 129 and 115 was found in DsOR and DbOR (Fig. 4C), respectively, and we constructed the mutants *DsOR^His^* (R129H) and *DbOR^His^* (R115H) for further overexpression experiments.

### DsOR interacted directly with DsPSY1/2.

To confirm the physical interaction between *Dunaliella* OR and PSY, we performed protein interaction analysis by the mating-based split ubiquitin system (mbSUS) of yeast two-hybrid assay (46), as OR was a transmembrane protein. The full-length *PSY* coding sequence was fused to NubG, a mutant of the N-terminal moiety of ubiquitin (Nub) with severely decreased affinity for the C-terminal moiety of ubiquitin (Cub), and *OR* was fused to Cub. As shown in Fig. 4D, all yeast transformants grew on synthetically defined (SD) medium lacking leucine, tryptophan, and methionine (SD/-Leu/-Trp/-Met), indicating that exogenous PSY are not toxic on yeast. Furthermore, yeast cells coexpressing NubG-DsPSY1/2 and DsOR-Cub could grow on SD medium lacking leucine, tryptophan, histidine, and adenine (SD/-Leu/-Trp/-His/-Ade) containing 75 and 400 μM methionine (Met), which was consistent with the positive control, but no growth was observed on the same media when there was coexpression with NubG-DsPSY1/2+Cub and NubG+DsOR-Cub, suggesting that DsOR could interact with DsPSY1/2. No growth was also observed on the same media when there was coexpression with NubG-DbPSY+DbOR-Cub, suggesting no interaction between DbOR and DbPSY, which may be due to the fact of the non-β-carotene-accumulating *Dunaliella* sp. FACHB-847 (25). Clearly, the mbSUS yeast two-hybrid assay demonstrated that DsOR interacted with DsPSY1 and DsPSY2 without significant difference, implying the key role of DsOR protein in regulating carotenoid metabolism in *D. salina*.

Previous study showed that two *Arabidopsis* OR proteins (AtOR and AtOR-like) as well as the *Brassica oleracea* OR protein (BoOR) interacted directly with PSY (12). Interestingly, differential interactions of OR proteins with the PSY enzymes were observed in saffron as CsOR-a displayed strong interactions with CsPSY1a, CsPSY1b, and CsPSY3, and weak interaction with CsPSY2, and in contrast, no interactions were observed between CsOR-b and the CsPSY enzymes (47). It was showed that the OR protein possessed an N-terminal region responsible for interaction with PSY and a C-terminal cysteine-rich zinc finger domain present in DnaJ-like molecular chaperones (12, 44, 47). Besides, OR protein has been found to interact with many other proteins, including carotenoid cleavage dioxygenase 4 (CCD4), oxygen-evolving enhancer protein 2-1 (PsbP, an extrinsic protein of photosystem II), eukaryotic release factor eRF1-2, plastid division factor ARC3, and other chaperones (45, 48 to 52), indicating its multifunctional roles.

### Heterologous expression of DsOR in carotenoid-producing E. coli.

Previous studies showed that overexpression of *OR*, especially *OR^His^*, genes in plants and green alga C. reinhardtii could significantly enhance the accumulation of carotenoids (16 to 18, 23). To investigate the effect of *Dunaliella* OR protein on PSY activity and the role of OR protein in regulating carotenoid metabolism, we coexpressed *Dunaliella OR* expressed *OR^His^* in E. coli carrying *crtEIY* and *Dunaliella PSY* genes. As shown in Fig. 2, for the expression of *DsOR* or *DsOR^His^* genes, the β-carotene contents of 07 dB+Y1-DsOR and 07 dB+Y2-DsOR had slight improvement compared with 07 dB+Y1 and 07 dB+Y2, and more β-carotene contents were detected in 07 dB+Y1-DsORH and 07 dB+Y2-DsORH, indicating that DsOR and DsOR^His^ can promote carotenoid accumulation, which was quite in line with the previous report (53). Interestingly, no significant change was observed in 07 dB+Yb-DbOR and 07 dB+Yb-DbORH compared with 07 dB+Yb, which may be due to the fact of the non-β-carotene-accumulating *Dunaliella* sp. FACHB-847 (25, 30). In addition, coexpression of *OR* or the *OR^His^* mutant in β-carotene-producing E. coli carrying *crtEIBY* genes also could not significantly affect the β-carotene levels compared with Ec07, suggesting that OR protein could not act on bacterial crtB, which was consistent with the previous report (53). Apparently, our study demonstrated that DsOR plays a crucial role in promoting carotenoid biosynthesis.

### Overexpression of *DsOR*/*DsOR^His^* enhanced β-carotene accumulation in *D. salina*.

**FIG 5:**
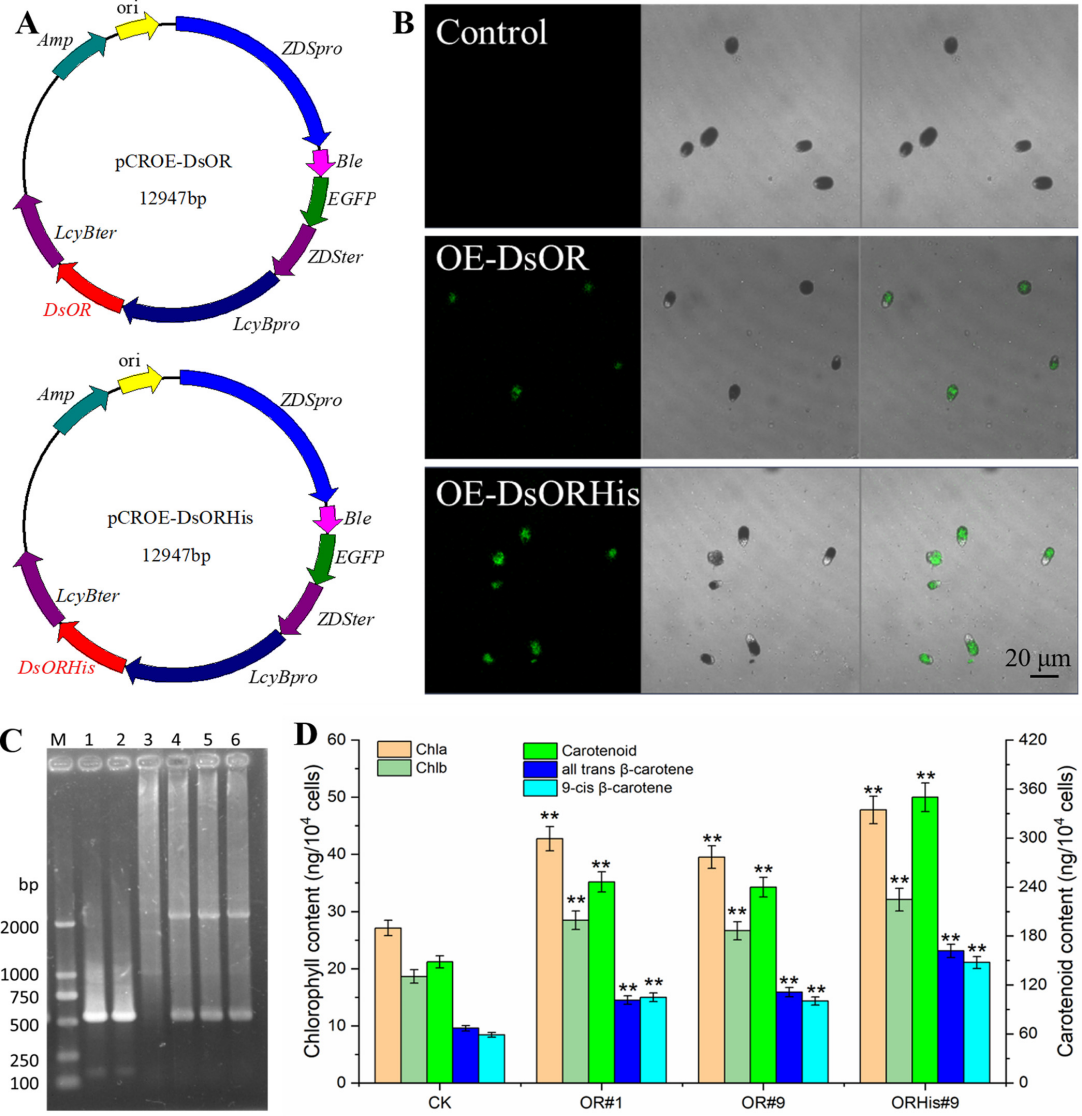
Overexpression of *DsOR* and *DsOR^His^* genes increased the single-cell carotenoid accumulation in *D. salina*. (A) Construction of overexpression vectors pCROE-DsOR and pCROE-DsORHis; (B) the transgenic algal cells observed by confocal laser scanning microscope; (C) verification of positive transformants by PCR using the algal genome DNA. Lane M, DL2000 Marker; 1, PCR product of plasmid pCROE-DsOR; 2, PCR product of pCROE-DsORHis; 3, PCR product of control algal genome DNA; 4 to 6, PCR products of the transformants DsOR#1, DsOR#9, and DsORHis#9, respectively. (D) Chlorophyll and carotenoid accumulation in the transformants at day 25.

By using efficient endogenous promoters in *D. salina* and the fusion of bleomycin-resistant gene *Ble* and the enhanced green fluorescent protein *EGFP* as reporter, the overexpression vectors, pCROE-DsOR, and pCROE-DsORHis, were constructed (Fig. 5A). The electrotransformation technology was used to transform these two overexpression vectors into *D. salina* cells, respectively. Three days after transformation, confocal laser scanning microscopy was used to observe the transgenic *D. salina* cells, and it was found that the cells after transforming the overexpressed vector showed green fluorescence (Fig. 5B), indicating that the overexpression vector was successfully transformed into *D. salina* cells. Meanwhile, bleomycin-resistant plates were used to screen the monoclonal algal transformants. Subsequently, two OE-DsOR strains (DsOR#1 and DsOR#9) overexpressing *DsOR* and one OE-DsORHis strain (DsORHis#9) overexpressing *DsOR^His^* were identified by direct PCR using algal cells as the template and confirmed by PCR using algal genomic DNA as the template (Fig. 5C).

**FIG 6:**
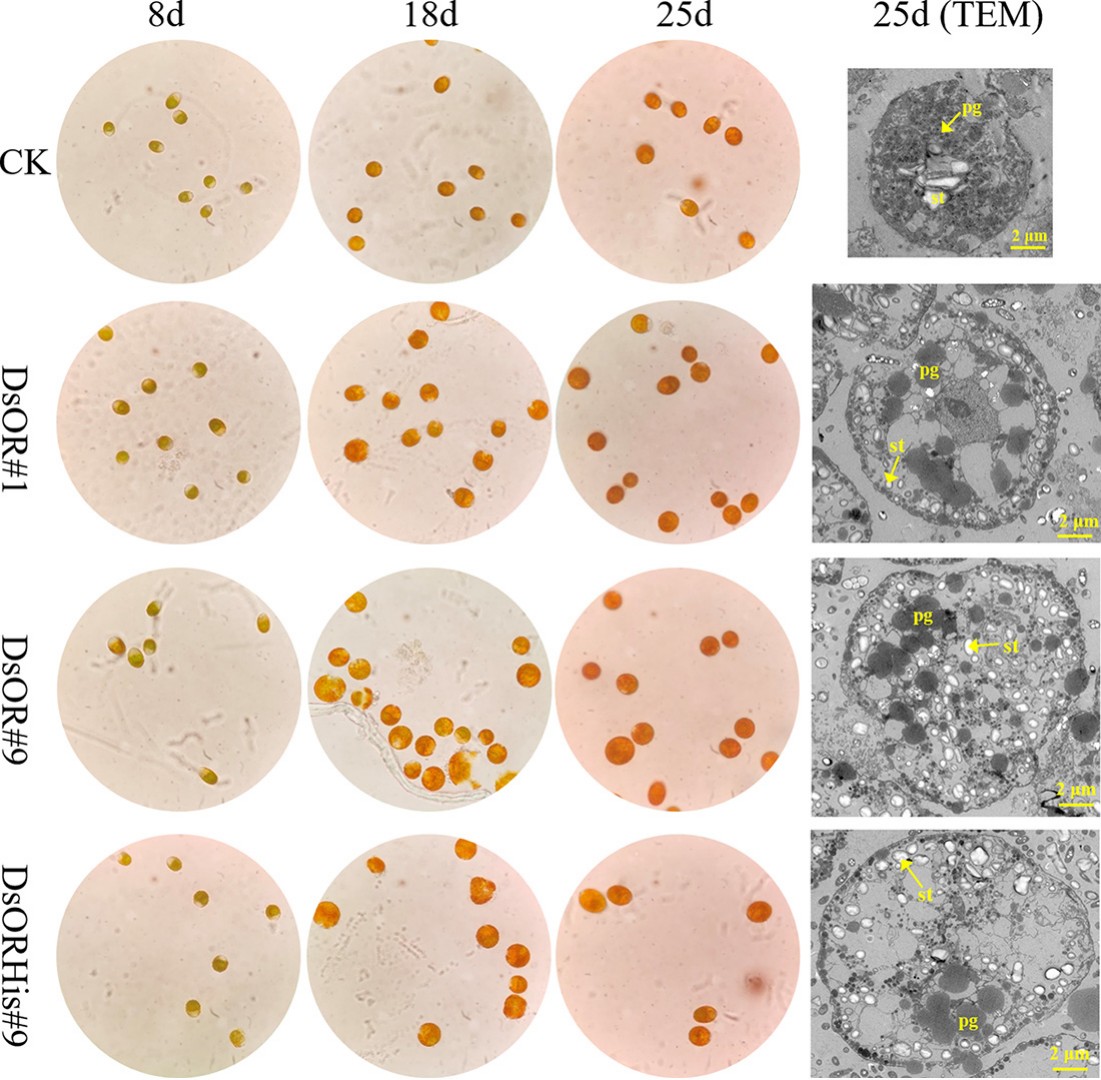
Effect of overexpression of *DsOR* and *DsOR^His^* genes on cell morphology of *D. salina*. Nontransgenic and transgenic *D. salina* cells were observed by light microscopy and transmission electron microscopy (TEM). pg, plastoglobuli (black dots); st, starch granules (white).

Phenotypic identification and carotenoid detection (Fig. 5D; Table S1 at https://www.biosynnatlab.com/wp-content/uploads/2023/03/Supporting-Information2023OR.pdf) showed that the chlorophyll and carotenoid contents per cell of the transformants at day 25 were higher than that of the control, of which DsOR#1 and DsOR#9 showed 22.9 to 30.7% higher single-cell carotenoids than the control, and DsORHis-9 had 73.4% higher than the control. *All-trans* β-carotene and *9-cis* β-carotene at the ratio of about 1:1 were the main carotenoids (accounting for >83%) in *D. salina* cells, which was consistent with a previous report (27). *D. salina* cells have turned red at day 25, and the carotenoid-to-chlorophyll ratio was 3.2 in the control *D. salina* cells and 3.5 to 4.4 in the transformants, which was in accordance with the previous report (54). Observed by light microscope and transmission electron microscope, all the transformants (DsOR#1, DsOR#9, and DsORHis#9) possessed larger and darker orange cells compared with the control, especially at the later stage of growth (Fig. 6). Interestingly, the transformants not only changed the cell size (enlarged cells), but also increased the size of plastoglobuli and changed the distribution of pyrenoid and starch granules (Fig. 6). Normally, orange *Dunaliella* cells contain a single cup-shaped chloroplast with uniform size of many plastoglobuli as well as a central pyrenoid surrounded by starch granules (55, 56). Due to the lack of a cell wall in *D. salina*, some cells tended to crack after enlarging (Fig. 6). The growth state of the transformants with low cell density was not as good as that of the control. This may be because the transformants with larger cells needed higher energy and more carbon sources, resulting in slightly slower cell growth. Also, pyrenoid is a subcellular structure that colocalizes CO_2_-concentrating mechanism and the ribulose-1,5-bisphosphate carboxylase/oxygenase (RuBisCo) enzyme, thus maximizing CO_2_ fixation in *Dunaliella* (56, 57). Therefore, the change in the distribution of pyrenoid and starch granules may influence the efficiency of CO_2_ concentration and photosynthesis. In conclusion, overexpression of the *DsOR* or *DsOR^His^* gene could significantly improve the single-cell carotenoid content of *D. salina*, with much more single-cell carotenoids in *D. salina* overexpressing *DsOR^His^*, which was well consistent with the *OR* Rubisco *OR^His^* transgenic lines of Chlamydomonas reinhardtii (23), tomato (17), and melon (18).

### Major conclusion.

In the β-carotene-accumulating *D. salina*, the efficient DsPSY1 played a dominant role in carotenogenesis, and DsPSY2 almost had no catalytic activity. Two key amino acid residues at positions 144 and 285 in the conserved domains were correlated with the functional variance between DsPSY1 and DsPSY2. DsOR could promote carotenoid accumulation via interacting with DsPSY1/2 and regulating plastid development, which provides important clues for the molecular mechanism of carotenoid metabolism regulation in *D. salina*. Overexpression of *DsOR* and *DsOR^His^* genes not only increased the single-cell carotenoid accumulation, but also enlarged cells, increased the size of plastoglobuli, and changed the distribution of pyrenoid and starch granules. In the lutein-rich *Dunaliella* sp. FACHB-847, DbPSY had more efficient catalytic activity than DsPSY1, but DbOR could not interact with DbPSY, which provides an explanation for the non-β-carotene overproduction in *Dunaliella* sp. FACHB-847. The rational design of highly efficient PSY, the regulatory factors or proteins for fine-tuned regulation of PSY expression, and the molecular mechanism of massive accumulation of β-carotene in *D. salina* need to be further explored.

## MATERIALS AND METHODS

### Algal strains and cultivation conditions.

*Dunaliella salina* CCAP 19/18 was obtained from the Culture Collection of Algae and Protozoa (CCAP), Scotland, United Kingdom. *D. salina* was cultivated in 2ASW (artificial seawater) medium containing 1.5 M NaCl (Table S2 at https://www.biosynnatlab.com/wp-content/uploads/2023/03/Supporting-Information2023OR.pdf). *Dunaliella* sp. FACHB-847 (formerly named *Dunaliella bardawil* strain FACHB-847) (29) obtained from the Freshwater Algae Culture Collection at the Institute of Hydrobiology (FACHB), Chinese Academy of Sciences, was grown in a *Dunaliella* medium as previously reported (58). Algal cells were cultivated at 26°C in a growth chamber and 8000 lx provided by cool-white fluorescent lamps under a 16/8 h light/dark cycle. The optical density (OD) of the algal sample was determined by using a spectrophotometer (Agilent, USA) at 630 nm (OD_630_). For algal biomass, the dry cell weight (DCW) of *D. salina* was calculated by OD_630_, with a formula y = 1794x–23.218, R^2^ = 0.999, where y = DCW (mg/L) and x = OD630 value (0.05 < OD_630_ < 1.0). Cell counts of the algal cultures were detected by using a Zeiss Axioplan microscope (Germany) equipped with a 40× (N.A. 0.75) plan-apochromatic objective and a 100-W mercury lamp with the hemocytometer. The genomic DNA of *Dunaliella* cells was extracted by EZNA HP Plant DNA kit (Omega Bio-Tek, USA).

### Gene mining and bioinformatics analysis.

Ten milliliters of algal cells collected at the late log phase were used for RNA isolation by RNAiso Plus (TaKaRa, Japan). Then, the complementary DNA (cDNA) was synthesized by RevertAid First Strand cDNA Synthesis kit (Thermo Scientific, USA). Genes from *Dunaliella* cells could be cloned by using the above cDNA template and PrimeSTAR HS DNA polymerase (TaKaRa, Japan), and primers (Table S3 at https://www.biosynnatlab.com/wp-content/uploads/2023/03/Supporting-Information2023OR.pdf) designed according to the genome or transcriptome data (31, 32).

Multiple sequence alignment was performed by ClustalX2 software. Conserved domains in protein were analyzed using the Conserved Domains Search tool (http://www.ncbi.nlm.nih.gov/Structure/cdd/wrpsb.cgi). Phylogenetic tree was conducted using the neighbor-joining method of MEGA7 software with the Jones-Taylor-Thornton substitution model, and robustness of the results was tested with 1,000 bootstrap replicates. Subcellular localization of protein was predicted by Plant-mPLoc (http://www.csbio.sjtu.edu.cn/bioinf/plant-multi/) and LOCALIZER (https://localizer.csiro.au/). Chloroplast transit peptide was predicted by the ChloroP 1.1 server (https://services.healthtech.dtu.dk/services/ChloroP-1.1/). Protein family was analyzed by Pfam (http://pfam.xfam.org/). Transmembrane helices and protein structure were predicted by Phyre2 (http://www.sbg.bio.ic.ac.uk/phyre2/html/page.cgi?id=index). The transit peptide and nuclear localization signal were predicted by LOCALIZER (https://localizer.csiro.au/). PolyPhen-2 (Polymorphism Phenotyping v.2, http://genetics.bwh.harvard.edu/pph2/) was used to predict possible impact of an amino acid substitution on the structure and function of a protein. Homology modeling was performed by SWISS-MODEL (https://swissmodel.expasy.org/interactive). All protein structures were visualized using VMD and SPDBV software. Superposition of two or more protein structures was performed using SuperPose v. 1.0 (http://superpose.wishartlab.com/) with RMSD (root mean square deviation) statistics.

### Heterologous expression of phytoene synthase and orange protein from *Duanliella* in E. coli.

The plasmid pACCAR16Δ*crtX* (named pAC-BETA here) harboring a carotenogenic gene cluster composed of *crtE*, *crtI*, *crtY*, and *crtB* from Pantoea ananatis (formerly named Erwinia uredovora) could be expressed in E. coli, and the resulting recombinant E. coli strain (named Ec07 here; Table S4 at https://www.biosynnatlab.com/wp-content/uploads/2023/03/Supporting-Information2023OR.pdf) led to the production of β-carotene (40, 59). In this study, the function of DsPSY1/2 was investigated by color complementation using the carotenoid-producing E. coli platform. First, the *crtB*-deleting fragment EIYdB was amplified by using premix PrimeSTAR HS DNA polymerase (TaKaRa, Japan), dcrtB For, and dcrtB Rev as primers (Table S3 at https://www.biosynnatlab.com/wp-content/uploads/2023/03/Supporting-Information2023OR.pdf), and pAC-BETA as the PCR template. The EIYdB fragment was subcloned into the EcoRV site of the pACYC184 vector (HonorGene, China) by using pEASY-Basic Seamless Cloning and Assembly kit (Beijing TransGen Biotech), to construct pAC-EIYdB, which was the *crtB*-deleting version of pAC-BETA. The CDS of *DsPSY1/2* or *DbPSY* without chloroplast transit peptide was subcloned into the *Asc*I-SalI site of the pCDFDuet-1 vector (Novagen, USA) to construct pCDF-Y1, pCDF-Y2, and pCDF-Yb (Table S4 at https://www.biosynnatlab.com/wp-content/uploads/2023/03/Supporting-Information2023OR.pdf). The *crtB*-deleting vector pAC-EIYdB, coupled with the plasmid-carrying exogenous or mutated *PSY* gene, was transformed in E. coli BL21(DE3) so as to determine the function and key amino acid residues affecting catalytic activity of *Dunaliella* PSYs. PCR-based site-directed mutagenesis was conducted as previously reported (60). The plasmids pCDF-Y1H, pCDF-Y1W, pCDF-Y2F, pCDF-Y2G, pCDF-YbH, pCDF-YbW, pCDF-DsORH, and pCDF-DbORH were generated, carrying *DsPSY1^F144H^*, *DsPSY1^G285W^*, *DsPSY2^H144F^*, *DsPSY2^W285G^*, *DbPSY^F155H^*, *DbPSY^G296W^*, *DsOR^His^* (R129H), and *DbOR^His^* (R115H). The plasmids pCDF-Y1HW, pCDF-Y2FG, and pCDF-YbHW carrying *PSY* genes with double mutations were also generated.

The CDS of *Dunaliella OR* or *OR^His^* was subcloned into the EcoRV-XhoI site of pCDFDuet-1 or vectors carrying the *PSY* gene (Table S4 at https://www.biosynnatlab.com/wp-content/uploads/2023/03/Supporting-Information2023OR.pdf). The plasmids carrying *PSY* and *OR* (or *OR^His^*) genes were coupled with 07 dB plasmid for mutational analysis by color complementation, so as to determine the role of OR in affecting carotenoid biosynthesis. The 30-mL overnight culture of the engineered E. coli strain was transferred into 120-mL LB medium with the appropriate antibiotics at 37°C, 220 rpm until the OD_600_ was 0.6. Then the culture was induced by 1.0 mM isopropyl β-D-1-thiogalactopyranoside (IPTG) for 48 h at 30°C, 200 rpm.

### Extraction and analysis of carotenoids.

Carotenoids were extracted from E. coli cells or algal cells as previously reported (25), and carotenoids were detected by high-performance liquid chromatography (HPLC) according to our previous reports (30).

### Mating-based split ubiquitin system (mbSUS) assay for protein interaction.

The interactions between *Dunaliella* OR and PSY were determined by mbSUS yeast two-hybrid as described previously (46). The *Dunaliella OR* gene was cloned into the HindIII-PstI site of the pMetYCgate vector (for fusion proteins with Cub) to construct the bait vectors pMetYCgate-DsOR and pMetYCgate-DbOR, and then transformed into the yeast haploid strain THY.AP4. Similarly, the coding sequences of *DsPSY1/2* and *DbPSY* without the stop codon were cloned into the EcoRI-SmaI site of pNXgate32-3HA (to express fusion proteins with Nub) to construct the prey vectors pNXgate32-DsPSY1/2-3HA and pNXgate32-DbPSY-3HA, respectively (for primers, see Table S3 at https://www.biosynnatlab.com/wp-content/uploads/2023/03/Supporting-Information2023OR.pdf). The bait vector and prey vector were then cotransformed into the yeast strain THY.AP4. Protein–protein interactions were tested by the growth of diploid yeasts on SD/-Leu/-Trp/-Met and SD/-Leu/-Trp/-His/-Ade with 0-, 75-, and 400-μM methionine (Met) at 30°C for 3 to 6 days.

### Transmission electron microscopy (TEM).

Ten milliliters of algal sample were centrifuged (3,500 *g*, 10 min), fixed overnight at 4°C using 2.5% glutaraldehyde, then postfixed in 1% osmium tetroxide for 2 h, and finally washed three times using phosphate-buffered saline (PBS). Subsequently, the samples were dehydrated with gradient ethanol solutions of 30%, 50%, 70%, 80%, 90%, 95%, and 100% for 15 min each time, then dehydrated with pure acetone for 20 min. After dehydration, the samples were embedded with epoxy resin. Ultrathin sections of 70 to 90 nm in thickness were cut using an ultramicrotome (EM UC7, Leica, Germany), then stained with lead citrate and uranyl acetate, and finally examined under a Hitachi transmission electron microscope (HT7700, Tokyo, Japan) at 75 kV.

### Overexpression of *DsOR*/*DsOR^His^* in *D. salina*.

For vector construction (primers seen in Table S3 at https://www.biosynnatlab.com/wp-content/uploads/2023/03/Supporting-Information2023OR.pdf), the fragments of *DsLcyB* (*lycopene β-cyclase* gene from *D. salina*) promoter (40), *DsOR* or *DsOR^His^* genes, and *DsLcyB* terminator were subcloned into the XhoI-XbaI site of the pZBET vector mentioned in our previous report, which carried the promoter and terminator of the *Dunaliella ξ-carotene desaturase* gene (*DbZDS*) and the fusion of *Ble-EGFP* as reporter (61), by the using pEASY-Basic Seamless Cloning and Assembly kit (Beijing TransGen Biotech). The overexpression vectors pCROE-DsOR and pCROE-DsORHis were then constructed. The electroporation procedure for introduction of foreign genes in *D. salina* cells and the selection of positive transformants were reported previously (40, 62). The primers (BE-For and BE-Rev) for selection of the positive transformants are shown in Table S3 (https://www.biosynnatlab.com/wp-content/uploads/2023/03/Supporting-Information2023OR.pdf). The fluorescent signal of EGFP in the transformants was observed by using confocal laser scanning microscope (ZEISS LSM710, Germany) after 3 days of transformation.

### Statistical analysis.

All experiments were performed in triplicate. Data were expressed as means ± SD (standard deviation). The significance of differences between groups was assessed by one-way analyses of variance or *t* test using IBM SPSS statistics 22. *P < *0.05 indicated the presence of a statistically significant difference, and *P < *0.01 was considered highly significant.
